# A robust single-beam optical trap for a gram-scale mechanical oscillator

**DOI:** 10.1038/s41598-017-15179-x

**Published:** 2017-11-06

**Authors:** P. A. Altin, T. T.-H. Nguyen, B. J. J. Slagmolen, R. L. Ward, D. A. Shaddock, D. E. McClelland

**Affiliations:** Centre for Gravitational Physics, The Australian National University, Science Rd 38a, 0200 Canberra, ACT Australia

## Abstract

Precise optical control of microscopic particles has been mastered over the past three decades, with atoms, molecules and nano-particles now routinely trapped and cooled with extraordinary precision, enabling rapid progress in the study of quantum phenomena. Achieving the same level of control over macroscopic objects is expected to bring further advances in precision measurement, quantum information processing and fundamental tests of quantum mechanics. However, cavity optomechanical systems dominated by radiation pressure – so-called ‘optical springs’ – are inherently unstable due to the delayed dynamical response of the cavity. Here we demonstrate a fully stable, single-beam optical trap for a gram-scale mechanical oscillator. The interaction of radiation pressure with thermo-optic feedback generates damping that exceeds the mechanical loss by four orders of magnitude. The stability of the resultant spring is robust to changes in laser power and detuning, and allows purely passive self-locking of the cavity. Our results open up a new way of trapping and cooling macroscopic objects for optomechanical experiments.

## Introduction

Optical springs have been proposed as a solution to the problem of mechanical coupling which impedes the operation of macroscopic objects in the quantum regime^1,2^. Purely optical trapping allows for extreme isolation of a mechanical oscillator from its environment and precise manipulation of the oscillator’s state via optical fields, facilitating cooling to the quantum ground state and the creation of entangled states with large numbers of particles^3–5^. In particular, levitation using optical springs is currently a topic of much research interest^6,7^, due to the potential of creating non-classical states of motion^8^. The optical spring effect is also important in interferometric gravitational wave detectors, in which the effect may be used to alter the frequency response of the interferometer and reduce the standard quantum noise limit around the spring resonance^9,10^.

Radiation pressure from a single circulating field in an optomechanical cavity gives rise to a static restoring force and anti-damping on the blue side of resonance, and conversely anti-restoring and damping forces on the red side of resonance^11–15^. Thus, a single-carrier system dominated by radiation pressure cannot be simultaneously both statically and dynamically stable. Several methods have been proposed to stabilize optical springs, most of which use two light fields, one at a large positive detuning providing a strong restoring force and weak anti-damping, and the other at small negative detuning providing strong damping and only weak anti-restoring. This technique was first demonstrated by Corbitt *et al*. in the LIGO Laboratory^15–17^, and was recently extended to use the two polarization modes of a birefringent cavity^18^. In addition to requiring two incident light fields, however, such methods work only at carefully chosen laser power and detuning, making them unsuitable for experiments in which these are critical tuning parameters.

Both thermoelastic and thermorefractive effects (known collectively as thermo-optic effects) can also modify the dynamic behavior of an optomechanical system^19–22^. In a normal Fabry-Perot cavity, the dominant thermo-optic effect is expansion of the mirror coating which reduces the effective cavity length. As demonstrated by Kelley *et al*. this tends to push the optical spring towards instability. It has been suggested that modified mirror coatings could achieve stability via thermo-optic feedback^23^, but this has yet to be shown experimentally.

Here, we demonstrate a robust single-beam optical trap for a macroscopic mechanical oscillator, using a modified optical spring which is both statically and dynamically stable, resulting in self-locking of the optical cavity. Cooling is achieved by rotating the optical spring quadrature using thermo-optic feedback, giving rise to a damping force several orders of magnitude stronger than the mechanical damping of the oscillator. This quadrature rotation produces a characteristic nonlinear dependence of damping on the cavity input power. We note that this effect is fundamentally different to the “photothermal pressure” observed in micro-mechanical oscillators^3,24–26^, in which thermal expansion applies a force to the oscillator which may partially cancel radiation pressure. In particular, since the damping in our experiment results from quadrature rotation of the optical spring, the dissipation is not thermal, but occurs via the (effectively) zero-temperature optical bath. In contrast to previous stabilized optical springs, our system is remarkably robust, exhibiting stability at any input power and any positive cavity detuning. Our work illustrates how even a small amount of absorption, an unavoidable and generally considered detrimental effect in high-quality optics, can be exploited to stabilize an optomechanical cavity, and opens up a new way of trapping and cooling macroscopic objects using purely optical forces.

## Results

### Displacement spectra

**Figure 1:**
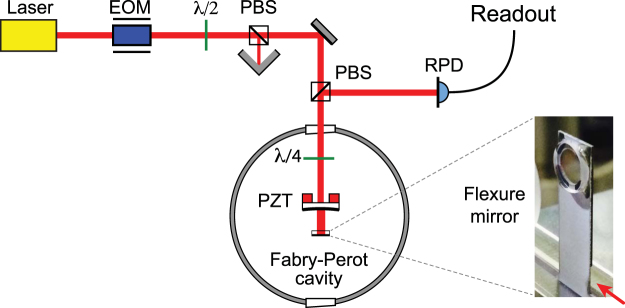
Schematic of the experimental setup (PBS: polarising beam splitter, EOM: electro-optic modulator, PZT: piezo-electric transducer, RPD: photodiode measuring reflected power). The thick black lines indicate the coatings on the cavity mirrors. The photo shows the rear mirror of the cavity mounted on the silicon flexure (red arrow).

Our experimental setup is shown schematically in Fig. 1. The mechanical oscillator comprises a high reflectivity 1/4″ dielectric mirror glued to a silicon flexure with a 100 *μ*m membrane. The oscillator has an effective mass of *m* = 0.3 g, a fundamental resonant frequency of 165 Hz (bending mode) and a quality factor *Q* = 55000 obtained from ringdown measurements. A single beam from a Nd:YAG laser at *λ* = 1064 nm is used to generate the radiation pressure force, provide the thermo-optic feedback, and read out the position of the flexure mirror. Stabilizing thermo-optic feedback is achieved by mounting the flexure mirror with the coating outside the cavity, such that thermorefraction in the mirror substrate and thermoelastic expansion of the coating both increase the effective round trip path length. This situation arises naturally in coupled cavity systems and coating-free mirrors^27–29^.

**Figure 2:**
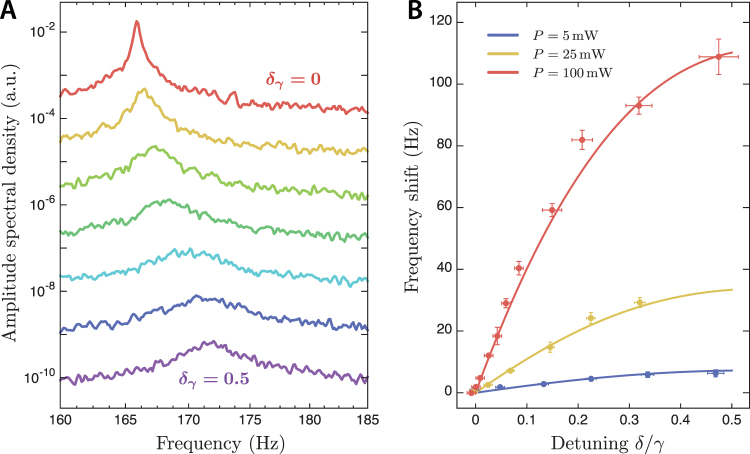
(**A**) Displacement spectra of the flexure for different cavity detunings on the blue side of resonance. The radiation pressure restoring force adds to the mechanical spring, increasing the flexure resonant frequency as the cavity is detuned by up to half a linewidth. Damping due to thermo-optic feedback is also visible as a broadening of the resonance peak. (**B**) Flexure resonant frequency as a function of cavity detuning for input powers of *P*_*in*_ = 5, 25 and 100 mW, obtained from fits to the displacement spectra. Error bars represent 95% confidence intervals in the fit parameter.

Figure 2 shows the displacement spectrum of the flexure around its mechanical resonant frequency for an input power of *P*_*in*_ = 25 mW and varying blue detuning of the cavity. Radiation pressure provides a static restoring force, stiffening the mechanical spring and increasing the observed resonant frequency. Simultaneously, the thermo-optic feedback damps the motion of the oscillator, broadening the resonance peak. For these measurements, the PDH error signal was used to lock the cavity to a particular detuning using a servo. The unity gain frequency of the servo was kept as low as possible (<10 Hz) to prevent the cavity from drifting away from resonance while having minimal influence on the mechanical motion of the flexure. However, servo feedback can mask the effect of thermo-optic feedback on the flexure’s dynamical response^30,31^; for this reason, all of the remaining experiments did not use servo feedback.

### Thermo-optic feedback model

The optical spring constant arises from the dependence of the cavity’s circulating power on the position of the moveable mirror, and is given by the following frequency-domain expression^13,16^

1 $$\begin{array}{c}{K}_{os}(\omega )=-\frac{2}{c}\frac{d{P}_{circ}}{dL}\\ \quad \quad \,\,\,\,\,=\frac{16\pi {P}_{in}{t}_{1}^{2}{r}_{1}{r}_{2}^{3}}{c\lambda {(1-{r}_{1}{r}_{2})}^{3}}\frac{{\delta }_{\gamma }}{1+{\delta }_{\gamma }^{2}}\frac{1+{\delta }_{\gamma }^{2}-{\omega }_{\gamma }^{2}}{{(1+{\delta }_{\gamma }^{2}-{\omega }_{\gamma }^{2})}^{2}+4{\omega }_{\lambda }^{2}},\end{array}$$

where *r*_*i*_ and *t*_*i*_ are the amplitude reflectivity and transmissivity, ${\omega }_{\gamma }\equiv \omega /\gamma$ and ${\delta }_{\gamma }\equiv \delta /\gamma$ are the frequency and detuning expressed in units of the cavity half-linewidth *γ*, and the Fabry-Perot response function is given by ${P}_{circ}={P}_{in}{t}_{1}^{2}{{\rm{(1}}-{r}_{1}{r}_{2})}^{-2}{\rm{/(1}}+{\delta }_{\gamma }^{2})$. Additionally, the circulating power lags behind the motion of the mirror due to the finite response time of the cavity, giving rise to a viscous damping force

2 $${{\rm{\Gamma }}}_{os}(\omega )=\frac{2{K}_{os}(\omega )}{m\gamma (1+{\delta }_{\gamma }^{2}-{\omega }_{\gamma }^{2})}{\rm{.}}$$

The short length *L* = 10 mm and relatively low finesse in our system result in a cavity decay rate $\gamma =2\pi \times 6.4$ MHz that far exceeds the mechanical frequencies of interest $\omega  \sim 2\pi \times 100$ Hz, thus we can take the DC values of the above expressions (${\omega }_{\gamma }\to 0$). The optical spring constant predicted by Equation (1) exceeds the flexure’s mechanical spring constant ${K}_{m} \sim 320\,$ Nm^−1^ at high input power, but the radiation-pressure-induced damping rate Γ_*os*_/2*π* ~ 1 mHz is negligible, and even lower than the mechanical damping Γ_*m*_ = *ω*_0_ /Q = 2*π* × 3 mHz. However, this is not the only source of optically induced viscous damping.

**Figure 3:**
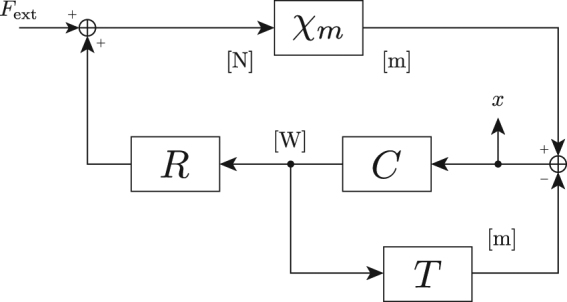
Block diagram of thermo-optic feedback in an optical spring. The effective mirror position *x* is read out via the cavity length measurement. The physical units at each point in the loop are shown in brackets.

In our system, both thermorefraction in the flexure mirror substrate and thermoelastic expansion of the substrate and coating tend to lengthen the cavity. Note that this change is in the same direction as that induced by radiation pressure. The block diagram in Fig. 3 shows how this affects the optical spring. In the presence of radiation pressure alone, the mechanical susceptibility ${\chi }_{m}^{-1}=-m{\omega }^{2}+{K}_{m}+im{{\rm{\Gamma }}}_{m}\omega$ gains an additional term ${K}_{os}=-CR$, where $C=d{P}_{circ}/dL$ is the cavity response and $R\,{\rm{=\; 2}}/c$ represents the radiation pressure force for a given optical power (c.f. Equation 1). Thermo-optic displacement acts as a feedback path affecting the cavity length directly, analogous to an electronic servo^30,31^. This has the effect of modifying the optical spring constant as follows:

3 $${k}_{opt}=\frac{{K}_{os}}{1+{G}_{T}},$$

where ${G}_{T}=CT$ is the complex open-loop gain of the thermo-optic feedback. Here we have taken only the real part of the optical spring constant since Γ_*os*_ is negligible in our system. However, the thermo-optic feedback can rotate *K*_*os*_ in the complex plane such that *k*_*opt*_ has a non-negligible imaginary part, which we observe as damping.

The thermo-optic feedback gain consists of the cavity response *C* to a change in mirror position, in units of W/m, and the thermo-optic response *T* to a change in absorbed power, in units of m/W, which we approximate with a low-pass filter response^32^. For the purposes of this model, we assume without loss of generality that absorption is evenly distributed throughout the mirror, such that thermorefraction dominates the thermo-optic response. In the Fourier domain:

4 $${G}_{T}(\omega )=CT=\frac{d{P}_{circ}}{dL}\frac{-\xi A}{1+i\omega {\tau }_{th}},$$

where *ξ* denotes the effective mirror displacement per unit absorbed power, *τ*_*th*_ is the thermal relaxation timescale, and *P*_*abs*_ = *AP*_*circ*_ with *A* the absorption coefficient. Higher-order corrections to this response have been calculated^23,32^ and even observed experimentally^33^, but this model suffices in the regime explored here. The relaxation time can be calculated from the thermal diffusion length to be ${\tau }_{th}=\rho C{r}_{0}^{2}/\kappa$ ^32^ and the cavity length change per unit power is $\xi =\beta /2\pi \kappa$, where $\beta =dn/dT$ is the thermorefractive coefficient^21,34^ and *ρ*, *C* and *κ* represent respectively the density, specific heat and thermal conductivity of the mirror coating, with *r*_0_ the 1/*e* ^2^ beam radius at reflection. For our fused silica substrate, with *ρ* = 2200 kg/m^3^, *C* = 746 J/kg/K, *β* = 8 ppm/K and *κ* = 1.38 W/m/K^34^, and a spot size of *r*_0_ = 129 *μ*m, we find *τ*_*th*_ = 10 ms and *ξ* = 920 nm/W. The absorption of the mirror was measured to be $A\simeq 30$ ppm.

### Spring constant and damping rate

**Figure 4:**
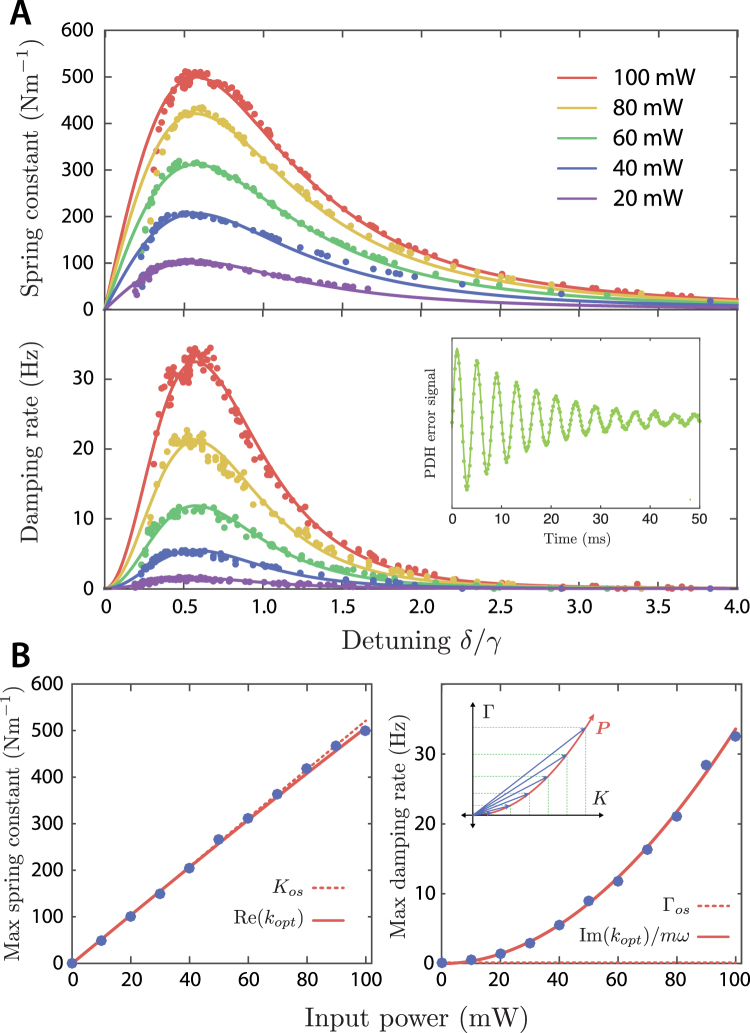
(**A**) Measured optical spring constant *K*_*opt*_ and damping rate Γ_*opt*_ as a function of cavity detuning, for various input powers. The lines are fits of Equation (3) to the experimental data, and the inset shows a typical ringdown measurement used to measure the frequency and damping rate. (**B**) Power dependence of the maximum spring constant and damping rate. The solid lines are the predictions of Equation (3), the dashed lines show the expected behavior in the absence of thermo-optic feedback. The nonlinear power dependence results from the feedback rotating the optical spring constant in the complex plane, as illustrated in the inset. Error bars are smaller than the data points.

Our system exhibits clear optically-induced damping on the same side of resonance as stiffening of the mechanical spring, as a result of thermo-optic feedback. This is in stark contrast to an optical spring dominated by radiation pressure, in which damping occurs at red detuning where the spring is softened, and stiffening is associated with anti-damping (dynamic instability). The full behaviour on the blue side of resonance is characterized in Fig. 4A, which shows the measured optical spring constant *K*_*opt*_ = *K*_*tot*_ − *K*_*m*_ and damping rate Γ_*opt*_ = Γ_*tot*_ − Γ_*m*_ as a function of detuning for different input powers. Each point is obtained from a fit of an exponentially decaying sinusoid to the observed ringing of the flexure as shown in the inset. With 100 mW input power, the maximum optical spring constant observed is (500 ± 10) Nm^−1^, which is more than 50% greater than the flexure’s mechanical spring constant. The solid lines are the predictions of Equation (3), and show excellent agreement with the data. We also observe instability when the cavity is red-detuned; this side of resonance would be expected to be both statically and dynamically unstable.

The power dependence of the optical spring constant and damping rate are illustrated in Fig. 4B. The spring constant is approximately linear in *P*_*in*_, while the damping is well described by a quadratic dependence. The model predictions are again in very good agreement with the data.

### Cavity self-locking

**Figure 5:**
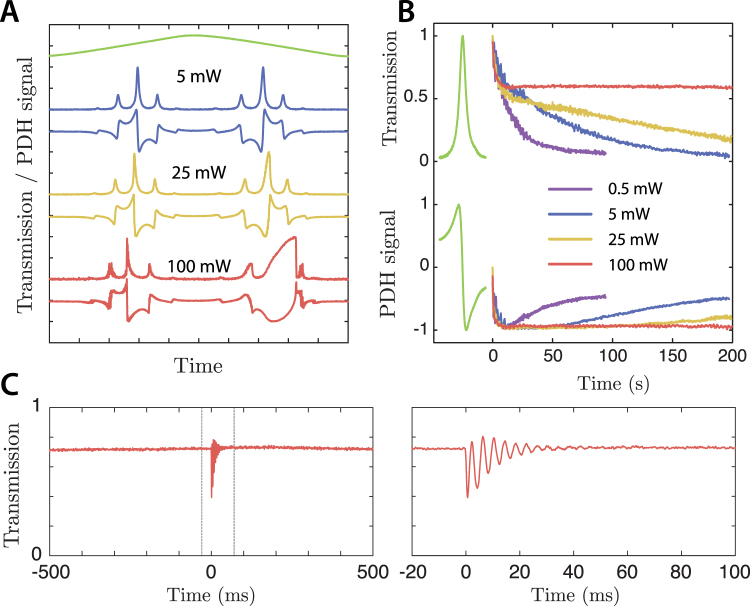
Cavity self-locking due to radiation pressue and thermo-optic feedback. (**A**) Cavity transmission and PDH error signal as the PZT voltage is swept slowly across resonance, for input powers of *P*_*in*_ = 5, 25 and 100 mW. (**B**) Self-locking of the cavity after being manually tuned to resonance via the PZT. At low input power the laser slowly drifts away from resonance, while at high power the optical forces keep the cavity at a blue detuning of $\delta /\gamma  \sim 0.5$. (**C**) Damping of oscillations caused by suddenly displacing the oscillator, demonstrating that a viscous damping force is present simultaneously with the restoring force that allows the cavity to self-lock.

Finally, we demonstrate that the thermo-optic feedback allows for fully stable self-locking of the cavity near resonance. Figure 5A shows the normalized cavity transmission and PDH error signal as the PZT voltage is swept slowly across resonance in both directions. At high power, the classic optical bistability behavior becomes apparent, with hysteresis visible between the two sweep directions. When placed near resonance manually using the PZT, the optical restoring force compensates for drifts in the laser frequency (Fig. 5B), and for an input power of 100 mW the cavity is observed to stay locked near a detuning of *δ*_*γ*_ = 0.5, with the transmitted power varying by only a few percent, for more than 1 hour. During this time, any sudden disturbance is quickly subdued by the thermo-optic feedback (Fig. 5C).

## Discussion

The real part of the observed spring constant displays the same ${\delta }_{\gamma }/{(1+{\delta }_{\gamma }^{2})}^{2}$ detuning dependence as expected from radiation pressure alone (Equation 1). This is expected for ${G}_{T}\ll 1$, since then $\Re ({k}_{opt})\simeq {K}_{os}[1-\Re ({G}_{T})]$ and the thermo-optic feedback is only a small correction to the radiation pressure spring constant. This correction is seen in the predicted power dependence of the spring constant (Fig. 4B), which begins to deviate from linear at high power.

The damping, on the other hand, depends on detuning as ${\delta }_{\gamma }^{2}/{(1+{\delta }_{\gamma }^{2})}^{4}$. This can be seen from Equation 4: for ${G}_{T}\ll 1$, $\Im ({k}_{opt})\simeq -{K}_{os}\Im ({G}_{T})$, and ${G}_{T}\propto \delta /{(1+{\delta }_{\gamma }^{2})}^{2}$ via the cavity response function. This also explains the quadratic power dependence of the damping rate, since both *K*_*os*_ and the thermo-optic feedback gain are proportional to *P*_*in*_. The nonlinear behavior in Fig. 4B is thus seen to be a consequence of the *K*_*os*_ vector being simultaneously lengthened and rotated in the complex plane, as depicted in the inset. We emphasize that this damping is not due to a photothermal force, but is caused by the modified optical spring, and thus dissipation occurs via the zero-temperature optical bath instead of via a thermal channel. Treating this effect as a photothermal force, as in ref.^3^, would predict a linear power dependence of the damping rate, which is clearly inconsistent with our observations.

The maximum observed damping rate ${{\rm{\Gamma }}}_{b}\,{\rm{ > \; 2}}\pi \times 30$ Hz exceeds the mechanical damping Γ_*m*_ by four orders of magnitude. In the present experiment, the displacement spectrum of the flexure is dominated by laser frequency noise, nonetheless we can calculate the minimum effective temperature that would be achievable if the system were limited only by optical damping. This is given by ${T}_{eff}/T={{\rm{\Gamma }}}_{m}{/{\rm{\Gamma }}}_{eff}\sqrt{{K}_{eff}/{K}_{m}}$ ^17^, which in our case is below 20 mK.

It is worth mentioning that although this damping is a result of loss in the cavity, very little absorption is required for thermo-optic feedback to stabilize the optical spring. Our mirrors were measured to have an absorption of 30 ppm, and the thermo-optic feedback gain (Equation 4) is linear in the absorption coefficient. Therefore, even a high-quality optic with only 1 ppm absorption would be expected to give a damping rate of ~1 Hz in our experiment.

## Conclusion

In conclusion, we have demonstrated a stable single-beam optical trap for a gram-scale mirror which is robust to changes in laser power and detuning. A restoring force induced by radiation pressure and damping generated by thermo-optic feedback combine create a stable optical trap and allow self-locking when the cavity is blue detuned. The optical damping is up to four orders of magnitude stronger than the mechanical damping in the system. A model of thermo-optic feedback with no free parameters shows excellent agreement with the observations. Our work represents the first experimental realization of a single-carrier optical spring stabilized by thermo-optic feedback, and contributes to the understanding of the interaction between thermo-optic effects and radiation pressure, with applications in systems with soft mechanical suspensions such as gravitational wave detectors. Our technique for creating a robust single-beam optical trap via thermo-optic feedback also offers a new way of achieving optical levitation of macroscopic objects, which is of interest for the preparation of non-classical states of motion.

## Methods

The mechanical oscillator comprises a high reflectivity 1/4″ dielectric mirror glued to a silicon flexure with a 100 *μ*m membrane. The silicon was laser-cut from a single wafer and the flexing membrane was machined using a Moore Nanotech 250 UPL ultra-precision lathe. The oscillator has an effective mass of $m=0.3$ g, a fundamental resonant frequency of 165 Hz (bending mode) and a quality factor *Q* = 55000 obtained from ringdown measurements. The mirror forms the back end of a Fabry-Perot cavity; the front mirror is mounted on a piezo-electric transducer (PZT) which we use to tune the cavity length. The cavity finesse is approximately ${\mathscr{F}}=1200$. The entire cavity is suspended by a four-stage vibration isolation system and placed inside a vacuum chamber evacuated to 10^−5^ mbar.

A single beam from a Nd:YAG laser at *λ* = 1064 nm is used to generate the radiation pressure force, provide the thermo-optic feedback, and read out the position of the flexure mirror. This beam is phase modulated at 76 MHz before entering the cavity to generate sidebands for readout using the Pound-Drever-Hall (PDH) technique^35^. The reflected beam is detected on a resonant photodiode and demodulated to provide an error signal which allows us to monitor the cavity length.

To measure the ringdown behavior of the flexure, a short pulse is applied to the PZT which excites oscillations due to a weak mechanical coupling between the cavity mirrors via the 2 kg steel cavity mount. These oscillations are visible both in the cavity transmission and the PDH error signal. Since the slope of the transmission is zero on resonance, and the slope of the error signal is zero at *δ* = *γ*, both measurements were needed to record the ringdown as a function of detuning (Fig. 4). For these measurements, the cavity detuning was set manually via a DC voltage applied to the PZT, but the laser and cavity were not actively stabilised. To account for variations in cavity detuning caused by relative fluctuations of the laser frequency and the cavity length, the actual cavity detuning for each ringdown measurement was calculated from the average transmission during the measurement period. The input laser power was measured outside the vacuum system using a power meter which is accurate to within 5%.
